# Hypothesis review: Alzheimer's overture guidelines

**DOI:** 10.1111/bpa.13122

**Published:** 2022-10-12

**Authors:** Isidro Ferrer

**Affiliations:** ^1^ Department of Pathology and Experimental Therapeutics University of Barcelona (UB) Barcelona Spain; ^2^ Neuropathology group Institute of Biomedical Research of Bellvitge (IDIBELL) Barcelona Spain; ^3^ Network Research Center of Neurodegenerative Diseases (CIBERNED), Instituto Carlos III Barcelona Spain

**Keywords:** Alzheimer's disease, genetics, human brain aging, membranes, neurofibrillary tangles, risk factors, senile plaques, tau, β‐Amyloid

## Abstract

National Institute on Aging–Alzheimer's Association definition and classification of sporadic Alzheimer's disease (sAD) is based on the assumption that β‐amyloid drives the pathogenesis of sAD, and therefore, β‐amyloid pathology is the *sine‐qua‐non* condition for the diagnosis of sAD. The neuropathological diagnosis is based on the concurrence of senile plaques (SPs) and neurofibrillary tangles (NFTs) designated as Alzheimer's disease neuropathological changes. However, NFTs develop in the brain decades before the appearance of SPs, and their distribution does not parallel the distribution of SPs. Moreover, NFTs are found in about 85% of individuals at age 65 and around 97% at age 80. SPs occur in 30% at age 65 and 50%–60% at age 80. More than 70 genetic risk factors have been identified in sAD; the encoded proteins modulate cell membranes, synapses, lipid metabolism, and neuroinflammation. Alzheimer's disease (AD) overture provides a new concept and definition of brain aging and sAD for further discussion. AD overture proposes that sAD is: (i) a multifactorial and progressive neurodegenerative biological process, (ii) characterized by the early appearance of 3R + 4Rtau NFTs, (iii) later deposition of β‐amyloid and SPs, (iv) with particular non‐overlapped regional distribution of NFTs and SPs, (v) preceded by or occurring in parallel with molecular changes affecting cell membranes, cytoskeleton, synapses, lipid and protein metabolism, energy metabolism, neuroinflammation, cell cycle, astrocytes, microglia, and blood vessels; (vi) accompanied by progressive neuron loss and brain atrophy, (vii) prevalent in human brain aging, and (viii) manifested as pre‐clinical AD, and progressing not universally to mild cognitive impairment due to AD, and mild, moderate, and severe AD dementia.

## Graphical abstract

Brain aging with ADNC and sporadic Alzheimer's disease (sAD) form a prevalent continuum unique to humans. Molecular changes affecting cell membranes, cytoskeleton, synapses, lipid and protein metabolism, energy metabolism, neuroinflammation, cell cycle, astrocytes, microglia, and blood vessels precede and occur in parallel with early ADNC; and they are drivers of sAD. Tau pathology is the first neuropathological hallmark but not necessarily the origin of the human brain aging with ADNC, and sAD.

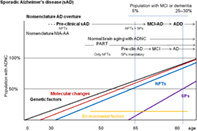

## 1 THE BEGINNING: PRE‐SENILE DEMENTIA, SENILE DEMENTIA, AND NORMAL BRAIN AGING WITH ALZHEIMER‐LIKE CHANGES

In the first decade of the 20th century, the use of the Bielchowsky silver staining permitted the discovery of neurofibrillary tangles (NFTs) and senile plaques (SPs) by Alois Alzheimer in a woman with pre‐senile dementia [1], and by Oskar Fischer in cases with senile dementia [2, 3].

Alzheimer's disease (AD), including pre‐senile and senile cases, was defined in 1984 as a neurodegenerative disease manifested by progressive dementia and characterized by brain atrophy, neuronal death, and a particular distribution of abundant SPs and NFTs in the brain [4].

NFTs in the hippocampus, entorhinal cortex, inferior temporal cortex, and, very rarely, the frontal neocortex, together with more variable presence and distribution of SPs, in old‐aged non‐demented individuals, were considered normal brain aging [5, 6, 7, 8].

In the early 90s, the Consortium to Establish a Registry for Alzheimer's Disease (CERAD) proposed a neuritic plaque score based on the number of SPs per mm^2^ and the individual's age as a predictor of dementia to distinguish normal brain aging from AD [9, 10]. NFTs were not considered in this score.

## 2 β‐AMYLOID AND TAU PATHOLOGY; FAMILIAL AND SPORADIC AD

In the middle 80s and early 90s, β‐amyloid was identified as the primary component of SPs and β‐amyloid cerebral angiopathy (Aβ‐CAA) [11, 12, 13, 14].

At the same time, abnormal tau protein was identified as the main component of NFTs [15, 16, 17, 18, 19]. Abnormal tau in AD comprises the six 3Rtau and 4Rtau isoforms resulting from *MAPT* (microtubule‐associated protein tau) splicing [20]. Abnormal tau also shows post‐translational modifications such as hyper‐phosphorylation, acetylation, glycosylation, and nitration. Reshaped tau conformation, truncation, oligomerization, and aggregation are added through the generation of NFTs [21, 22, 23, 24, 25, 26].

Mutations in *APP* (β‐amyloid precursor protein), *PSEN1* (presenilin1), and *PSEN2* (presenilin2) were causative of early‐onset familial Alzheimer's disease (EOFAD, or fAD) in about 10%–15% of early‐onset AD (EOAD) cases. *APP*, *PSEN1*, and *PSEN2* encode membrane proteins, and all are involved in producing β‐amyloid through the cleavage of APP by the combined action of β‐ and γ‐secretases. Increased APP dosage was also causative of fAD and β‐amyloid angiopathy [27, 28, 29, 30, 31, 32]. However, mutations in *MAPT* do not give rise to AD.

These discoveries led to the β‐amyloid cascade hypothesis, which supports the concept that the production of β‐amyloid fibrils is the primary factor triggering NFT formation and AD progression [33]. Later, the harmful effect of β‐amyloid was extended to β‐amyloid oligomers in addition to fibrils [34, 35].

Transgenic mice bearing only pathogenic *APP*, *PSEN1*, and *PSEN2* mutations develop cerebral and vascular β‐amyloidosis. Among these are APP/PS1 double Tg mice expressing a chimeric mouse/human amyloid precursor protein (Mo/HuAPP695swe) and a mutant human presenilin 1 (PS1‐dE9); and 5XFAD Tg mice over‐expressing mutant human amyloid beta (A4) precursor protein 695 (APP) with the Swedish (K670N, M671L), Florida (I716V), and London (V717I) fAD mutations along with human presenilin 1 (PS1) harboring two fAD mutations, M146L and L286V, but not NFTs; tau pathology is restricted to dystrophic neurites of SPs in transgenic mice. However, Tg mice bearing β‐amyloid‐related mutations and tau gene mutations develop SPs and NFTs, as in 3xTg‐AD (APPSwe,tauP301L) and (APPSwe,tauP301L1Lfa *Psen1* ^*tm1Mpm*^). Therefore, transgenic mouse‐bearing mutations linked to fAD are models of β‐amyloidopathy but not AD. Adding *mapt* mutations in the β‐amyloidopathy transgenic murine models is necessary to produce neuropathology similar to that seen in fAD.

Moreover, not all *APP* mutations causative of (Aβ‐CAA) and cerebral amyloidosis are accompanied by tau pathology as in the Dutch and Flemish inherited Aβ‐CAA. *APP* mutations causing primary cerebral hemorrhages are principally located within the Aβ domain [36].

Furthermore, the *γ*‐secretase complex may act on more than 90 substrates [37, 38]. The diversity of substrates at the cell membrane suggests that mutations in presenilin genes trigger β‐amyloid processing and may affect other membrane‐associated proteins. In this line, the *PSEN‐1* M146L mutation is causative of fAD with Pick bodies [39]. A patient with a familial history of early‐onset frontotemporal lobar degeneration carried the *PSEN‐1* M146V mutation; the post‐mortem neuropathological study disclosed β‐amyloid plaques, NFTs, Pick bodies in the hippocampus and cortex, cortical globose tangles, and ubiquitin‐positive nuclear inclusions in white matter oligodendrocytes [40]. The Gly183Val mutation *in PSEN1* is associated with Pick's disease but not β‐amyloid plaques [41].

In summary, APP, presenilin1, and presenilin 2 are involved in various cellular functions linked to cell membranes. It can be suggested that mutations in fAD genes may lead to complex membrane dysfunction beyond β‐amyloidogenesis. Altered membrane structure and function may facilitate tau phosphorylation and disrupt many metabolic signals. Cellular models learn about alternative or complementary dysfunctional consequences of AD‐linked mutated proteins and variants [42, 43, 44, 45].

However, about 95% of patients with dementia due to AD are sporadic (sAD), and mostly they are older than those suffering from EOAD (late‐onset Alzheimer's disease: LOAD). Genetic factors play variable roles in the genesis of sAD. Individuals with Down syndrome, caused by the presence of all or part of the third copy of chromosome 21, have large numbers of SPs and NFTs at the age of 40. Allele ε4 of apolipoprotein E (APOE) was the first identified low‐penetrating genetic risk factor of sAD [46, 47, 48]. Currently, more than 70 genetic risk factors have been identified using genome‐wide association studies in patients with clinical manifestations of sAD [49, 50, 51, 52, 53, 54, 55]. The products of these genes modulate lipid metabolism and cell membranes, cytoskeleton, and neuroinflammation [56]. A few gene variants appear to be involved in APP metabolism, mainly through their putative impact on membrane structure and protein cleavage.

Shockingly, tau pathology in sAD has also been considered a secondary tauopathy subjected to the driving forces of β‐amyloid pathology following the amyloid cascade hypothesis.

## 3 TAU AND β‐AMYLOID PATHOLOGY IN BRAIN AGING AND SAD

In the 90s, the systematic analysis of NFTs and SPs in post‐mortem brains of non‐demented and demented individuals revealed the natural distribution and progression of NFTs and SPs with age [57, 58, 59, 60, 61].

At cortical Braak and Braak stages I and II, NFTs appear in the entorhinal and transentorhinal cortex. At stages III and IV, NFTs progress to the hippocampus, temporal cortex, and limbic system nuclei. At stages V and VI, NFTs spread to most areas of the neocortex. The spreading of NFTs is accompanied by a dramatic increase in neurons with NFTs across stage progression [57, 58, 59, 60, 61]. The olfactory bulb and tract, and several nuclei of the brain stem, including the raphe nuclei and the locus coeruleus, are also affected by tau pathology at the first NFT cortical stages; the number of NFTs increases in these regions with the progression of the neurodegenerative process [62, 63, 64, 65, 66]. The occurrence of NFTs in selected brain stem nuclei is categorized as subcortical stages a‐c [61].

The distribution of SPs differs from NFTs in brain aging and sAD [67]. Stages 0, A, B, and C of Braak define the progression of SPs in the neocortex. Stage A: low density of SPs, especially in the frontal, temporal and occipital cortex; stage B: SPs in the neocortical association areas and hippocampus; stage C: in primary sensory and motor areas [57, 59, 61]. Thal's proposal categorizes phase 1: exclusively neocortex; phase 2: also allocortex; phase 3: diencephalic nuclei, striatum, and cholinergic nuclei of the basal forebrain; phase 4: brain stem; and phase 5: also the cerebellum [67].

The time of appearance of NFTs also differs from that of SPs. NFTs are identified in specific brain regions in young people in their twenties. The number of NFTs increases with age and affects about 85% of human beings at the age of 65, at least restricted to NFT stages I–III. About 98% of individuals have NFTs in the telencephalon at 80 [60, 61, 63, 68, 69]. In contrast, only about 30% have SPs at age 65 [57, 59, 61, 68, 69], and in around 60% over 80 [61]. NFTs without SPs are detected in about 35% of individuals older than 90 [61, personal observation].

The lack of temporal and regional concordance between NFTs and SPs is intuitively hardly consistent with the β‐amyloid cascade hypothesis in brain aging and sAD [70, 71]. However, this scenario does not contradict the evidence that tauopathy is fueled by amyloid precursor protein dysfunction [72, 73].

AD is unique to humans. Other species may have scattered β‐amyloid deposits and tau pathology, but in none of these do SPs and NFTs show the prevalence, localization, and widespread distribution they manifest in human beings. Even so, the presence of tau pathology and β‐amyloid deposits in certain old‐aged animals points to a link between brain aging and abnormal tau and APP metabolism in many species, including dogs, bears, pinnipeds, primates, and cetaceans.

## 4 REDEFINITION OF s AD IN THE SECOND DECADE OF THIS CENTURY: NATIONAL INSTITUTE ON AGING–ALZHEIMER'S ASSOCIATION GUIDELINES

Clinical and post‐mortem neuropathological sAD progression suggests a concatenation of Alzheimer's disease neuropathological changes (ADNC, covering SPs and NFTs) in sAD [57, 58, 59, 60, 61, 69, 74, 75, 76, 77, 78, 79, 80]. This situation prompted a clinical redefinition of AD at the beginning of the second decade of this century by the National Institute on Aging–Alzheimer's Association (NIA‐AA).

Three pillars underlay this new approach: (a) the neuropathological evidence of ADNC; (b) biochemical and neuroimaging biomarkers; and (c) clinical symptoms.

### 4.1 Neuropathology

NIA‐AA guidelines considered SPs and NFTs essential neuropathologic features of AD [81, 82]. The main points were (1) the recognition that ADNC may occur in the apparent absence of cognitive impairment; (2) the consideration of an “ABC” score for ADNC, incorporating histopathologic assessment of β‐amyloid deposits (called A, based on Thal phases), staging of NFTs (called B, based on Braak stages), and scoring of neuritic plaques (called C, based on CERAD); and (3) the assessment of co‐morbid conditions such as Lewy body disease, vascular brain injury, hippocampal sclerosis, and TDP‐43 proteinopathy that may modify the clinical presentation in every particular individual.

NIA‐AA guidelines assume that the appearance of SPs is the *sine‐qua‐non* condition for the neuropathological diagnosis of sAD. The presence solely of NFTs is not considered a prime manifestation of sAD [81, 82]. This way of thinking is based on the β‐amyloid cascade hypothesis as the origin and trigger component of AD.

### 4.2 Biomarkers

Current cerebrospinal fluid (CSF), plasma, and blood biomarkers used in AD diagnosis are β‐amyloid species, phospho‐tau and tau, phospho‐tau ratio, neurofilaments, synaptic proteins, activated astrocytes, and inflammatory markers [83, 84, 85, 86, 87, 88, 89, 90]. The available methods cannot detect differential levels of tau, phospho‐tau, β‐amyloid, and structural or synaptic proteins unless the degenerative process is at least at the middle stages of ADNC (A2, B2, C2, following the ABC score).

CT and MRI reveal that hippocampal atrophy is a late marker of AD that is only positive when there is advanced NFT pathology and neuron loss in the hippocampus. ^18^F‐Fluorodeoxyglucose *positron emission tomography* (^18^F‐FDG *PET*) and fMRI may detect hypo‐perfusion and hypo‐metabolism linked to neuronal function.

PET using specific radiotracers permits the visualization of abnormal protein deposits, particularly β‐amyloid and P‐tau species. A recent meta‐analysis revealed that ~25%–35% of cognitively normal older adults harbored a significant amount of β‐amyloid [91].

Tau‐PET shows early tau deposition in the entorhinal and temporal cortices in β‐amyloid‐negative non‐demented individuals and its progression to other brain regions following more advanced NFT Braak stages in individuals with added β‐amyloid pathology [92, 93, 94, 95, 96, 97, 98, 99]. Tau‐PET is considered a promising tool for better prediction of cognitive change than amyloid‐PET and MRI, and it may support the prognostic process in the pre‐clinical stages of AD [100].

PET studies, and particularly tau‐PET observations, confirm that: (i) tau pathology precedes by several decades the appearance of β‐amyloid in brain aging without cognitive impairment; (ii) tau pathology may be found in some individuals suffering from cognitive impairment without concomitant β‐amyloid deposition, and; (iii) tau pathology, rather than β‐amyloid pathology, correlates with progressive cognitive decline in sAD.

### 4.3 Clinical classification of Alzheimer's disease

A critical historical misunderstanding regards the term Alzheimer's disease (AD) as synonymous with Alzheimer's dementia. However, a significant achievement in understanding AD as a clinically progressive neurodegenerative process was formalized at the beginning of the second decade of this century by the NIA‐AA. Clinically AD was categorized as pre‐clinical AD, MCI due to AD, and mild, moderate, and severe Alzheimer's dementia [83, 101, 102, 103, 104, 105, 106, 107, 108, 109, 110, 111, 112, 113, 114, 115, 116, and https://www.alz.org/media/Documents/Alzheimer's-facts-and-figures].

Pre‐clinical AD is considered in individuals with measurable brain changes revealed by biomarkers that indicate the earliest signs of AD but have not yet developed symptoms such as memory loss. MCI due to AD is considered in people with biomarker evidence of ADNC plus new but subtle signs such as memory, language, and thinking problems. Pre‐clinical AD is contemplated as a biological situation that makes possible, but not obligatory, the appearance of dementia later in life in the context of ADNC.

The selection of biomarkers by the NIA‐AA is in line with the creed of the β‐amyloid cascade hypothesis. Pre‐clinical stage 1 is characterized by primary amyloidosis and assessed by the positivity of β‐amyloid biomarkers and negativity of tau biomarkers. Stage 2 is asymptomatic cerebral amyloidosis plus “downstream” neurodegeneration based on high CSF tau/P‐tau ratio, neuronal dysfunction, cortical thinning, and hippocampal atrophy. Pre‐clinical stage 3 is distinguished by cerebral amyloidosis, neurodegeneration, and subtle cognitive decline.

The early presence of positive tau‐PET in the inner regions of the temporal cortex in the absence of positive β‐amyloid markers does not merit the categorization of pre‐clinical AD according to the current definition of the NIA‐AA.

However, cognitive status correlates with NFT burden rather than β‐amyloid plaques [107].

## 5 PRIMARY AGE‐RELATED TAUOPATHY

The term “primary age‐related tauopathy” (PART) was coined to include cases with NFT pathology at stages I–IV of Braak in the absence of β‐amyloid plaques [108]. Patients are cognitively “normal for age” or maybe suffer from MCI; dementia is rare [108, 109, 110, 111]. Early tau pathology without β‐amyloid deposits can be detected by tau‐PET, thus allowing a clinical identification of the pathology during life [96, 99, 100]. PART is predominant until the age of 60–70, prior to the progressive appearance of SPs in the brain. At this point, the incidence of AD increases at the expense of reduced incidence of PART. Thus, AD prevails in individuals aged 80–90, whereas NFT‐only pathology accounts for about 20% of the population. Dementia only with tangles (or tangle‐predominant dementia), which would be the logical progression of PART, is very uncommon [112].

Genetic studies carried out in neuropathologically‐verified PART cases have shown a lower prevalence of *APOEε4*, rs28834970 *PTK2B*, rs6733839 in the *BIN1*, and *CR1* genes, and a higher prevalence of APOEε2 [110, 113, 114]. In contrast, tangle‐predominant dementia has been associated with *the MAPT H1* haplotype [115]. The proposal of PART as a new tauopathy is not widely accepted; PART is also interpreted as part of AD [61, 116, 117].

## 6 BRAIN ALTERATIONS IN THE AGING FRONTAL CORTEX AND SAD ARE NOT RESTRICTED TO NFTs AND SPs, AND THEY MAY PRECEDE ADNC

Molecular changes in brain aging and sAD are not restricted to β‐amyloid and abnormal tau accumulation. Multiple systems are primarily dysfunctional or secondarily damaged by abnormal β‐amyloid and tau species. Added molecular changes compromise: (i) synapses; neurotransmitters, neuromodulators, and related receptors, including acetylcholine and acetylcholine receptors, glutamate and glutamate receptors, γ‐aminobutyric acid (GABA) and GABA receptors, serotonin and 5‐hydroxytryptamine (5‐HT) receptors, noradrenergic system, adenosine receptors, endocannabinoids, cannabinoid receptors, endorphins, and orexin; (ii) trophic factors and receptors; (iii) mitochondria and oxidative phosphorylation; (iv) oxidative and nitrosative stress damage to lipids, nucleic acids, and proteins; (v) mitochondria/endoplasmic reticulum interactions; (vi) endoplasmic reticulum stress; (vii) failure of the ubiquitin‐proteasome system and autophagy to remove debris; (viii) granulovacuolar degeneration; (ix) purine metabolism; (x) histone modifications, DNA methylation, and hydroxymethylation; (xi) non‐coding RNAs; (xii) protein synthesis; (xiii) altered cell cycle and re‐entry; and (xiv) cell death. Other key elements in the pathogenesis of brain aging and sAD are early dysfunctional astrocytes and microglia, followed by altered oligodendrocytes; and alterations in the neurovascular system manifested as early reduction of the cerebral blood flow and abnormal blood barrier function. All these factors, together with β‐amyloid and tau, contribute to neuronal cell death and reduced neuronal connectivity [69, 118, 119, 120].

Notably, several of those determining molecular changes linked to brain aging and sAD precede the appearance of NFTs and SPs, as demonstrated by their occurrence in brain regions not affected by SPs and NFTs at NFT stages I and II. Molecular changes progress with particular profile‐, time‐, and region‐dependent patterns [120]. Molecular changes have deleterious effects on brain functions, involving various structures and pathways. Most of them may participate in the later development of tau pathology and β‐amyloid production [120].

NFTs and β‐amyloid, in turn, potentiate all the above‐mentioned molecular alterations, thereby creating positive feedback for the degenerative process [120]. Other putative factors influencing brain aging and sAD, categorized as environmental factors, include oral cavity infections, intestinal microbiota, intellectual reserve, diet, and good health [120].

Table 1 summarizes molecular changes in the frontal cortex and hippocampus in sporadic cases at NFT stages I and II.

**TABLE 1:** Altered components, pathways, and functions in the frontal cortex and hippocampus at NFT stages I and II preceding the appearance of NFTs and SPs in these regions in cases with pre‐clinical AD

Main components	Main functional effects
Aberrant cell‐cycle re‐entry and altered adult neurogenesis	Programmed cell death, activation of kinases, oxidative stress damage, tau hyperphosphorylation, activation of β‐amyloid pathways, altered NGF/proNGF/p75 signaling
Brain lipids	Progressive decrease in the levels of cholesterol, phosphatidylethanolamine, phosphatidyl inositol, phospholipid, ethanolamine plasmalogen, and sphingomyelin; progressive modifications in the composition of PUFAs, and higher levels of MUFAs: altered brain composition, altered cell signaling, altered neuroinflammatory responses Modifications in DHA, AA, and PUFAs produce an imbalance between their protective role (the adaptive responses derived from their lipid mediators) and a deleterious role (derived from their susceptibility to oxidation) Cholesterol‐derived lipid mediators, including 24‐ and 25‐hydroxycholesterol, produce apoptosis Increased lipid peroxidation results in altered membrane function Increased lipofuscin
Lipid rafts and cell membranes	Altered lipid raft composition involving plasmalogens, PUFAs (especially DHA and AA), total polar lipids (mainly phosphatidylinositol, sphingomyelin, sulfatides, and cerebrosides), and total neutral lipids (particularly cholesterol and sterol esters) alter membrane composition and impair normal cell membrane signaling Altered cell membrane composition impacts cytoskeletal proteins through protein–protein interactions, electrostatic interactions with lipid membranes, and lipid tails Increment in local cholesterol increases BACE1/AβPP interaction and facilitates the production of β‐amyloid Microglial pro‐inflammatory mediators generate membrane damage
Specialized membranes	Altered synaptic membranes Altered expression of certain neurotransmitter receptors and modulators of neurotransmission Altered connectivity
Mitochondria	Altered mitochondrial membranes Altered OXPHOS Increased production of ROS Altered mitochondrial DNA methylation Impaired cross‐talk between endoplasmic reticulum and mitochondria: altered MAM interaction
Oxidative stress damage	Mitochondria, peroxisomes, ER, microsomes, nucleus, and plasma membrane are potential sources of ROS Oxidative stress damage DNA, RNA, carbohydrates, lipids, and proteins
Protein synthesis impairment	Alterations of protein synthesis pathways at the level of the nucleolus, mRNAs, miRNAs, ribosomal proteins
Dysregulated protein phosphorylation	Dysregulated phosphoproteins at NFT stages I and II are membrane proteins; proteins of the cytoskeleton; proteins of the synapses and dense core vesicles; proteins linked to membrane transport and ion channels; kinases; proteins linked to DNA and protein deacetylation; proteins linked to gene transcription and protein synthesis, and proteins involved in energy metabolism Altered phosphorylation of selected proteins, accomplished by activation of several kinases, may alter membrane and cytoskeletal function, among these synaptic transmission and membrane/cytoskeleton signaling, in addition to energy metabolism, protein synthesis, and DNA homeostasis
Inflammation	Aging is accompanied by low levels of activated innate inflammatory responses Activated microglia showing increased expression of ApoE, triggering receptor expressed on myeloid cells 2 (TREM2), and lipoprotein lipase (LP2) Modified astrocytes: increased expression of glial fibrillary acidic protein (GFAP), S100β, and vimentin, and modifications in morphology and number Senescent astrocytes; senescence‐associated secretory phenotype manifested by increased production of pro‐inflammatory cytokines together with oxidative damage and increased superoxide production Early dysregulation of selected inflammatory mediators such as C3AR1, CSF1R, CSF3R, IL6, IL6ST, TGFB1, and IL10RA Different inflammatory responses occur simultaneously in different regions in the same individual Clinical evidence of the protective role of non‐steroidal anti‐inflammatory drugs at pre‐clinical stages of AD
Primary alteration of small cerebral blood vessels	Altered endothelium, pericytes, composition of basal membranes, and altered function of podocytes Impaired CBF Impaired glucose uptake Impaired BBB

Abbreviations: AA, arachidonic acid; BBB, blood–brain barrier; C3AR1, complement component 3a receptor 1; CBF, cerebral blood flow; CSF1R: colony‐stimulating factor 1 receptor; CSF3R, colony‐stimulating factor 3 receptor; DHA, docosahexanoic acid; IL10RA, interleukin‐10 receptor; IL6, interleukin‐6; IL6ST, interleukin‐6 signal transducer; MAM, mitochondria‐associated ER membranes; MUFAs, monounsaturated fatty acids; OXPHOS, mitochondrial oxidative phosphorylation system; PUFAs, polyunsaturated fatty acids; ROS, reactive oxygen species; TGFB1, transforming growth factor‐A1.

## 7 TOWARDS AN ALTERNATIVE INTERPRETATION OF BRAIN AGING AND s AD: A NEW DEFINITION OF AD

The natural history of ADNC changes during the human lifespan shows the early formation of NFTs, followed by the appearance of β‐amyloid deposits decades later [57, 58, 59, 60, 61, 69]. This fact has been interpreted in two ways. Defenders of the β‐amyloid cascade hypothesis, represented by the NIA‐AA, split the process into two diseases: AD and PART. Other researchers postulate that tau pathology is an initiating factor in sAD [61]. However, there is no 3R tauopathy or 4R tauopathy and no other sporadic 3R + 4R tauopathy linked to the generation and deposition of β‐amyloid. Therefore, it is speculative to posit that tau pathology sets off β‐amyloid production in sAD, even though NFTs are the first ADNC in human brain aging [61].

Another proposal suggests the existence in the human brain of a PART to which β‐amyloid deposition is added in a time‐, rate‐ and region‐dependent manner in the different AD categories, reliant on genetic factors involved in the production of β‐amyloid [121].

Not surprisingly, the mutually exclusive hypotheses formulated to explain sAD are not satisfactory and have not produced significant beneficial results when applied in clinical trials. In short, anti‐β‐amyloid therapies have been unsuccessful not because they cannot reduce β‐amyloid deposits, as they indeed do, but rather because they do not stop the progression of the disease. In addition, therapies geared to reducing abnormal tau deposits face difficulty picking up the critical tau species, which may block NFT formation. Moreover, current therapies do not contemplate actions directed to the multiple genetic and molecular factors, which are indeed the inducers of ADNC.

A new concept and definition of brain aging and sAD are brought forward for further discussion. AD overture guidelines advance that sAD is: (i) a multifactorial and progressive neurodegenerative biological process, (ii) characterized by the early appearance of 3R + 4Rtau NFTs, (iii) later deposition of β‐amyloid and SPs, (iv) with particular non‐overlapped regional distribution of NFTs and SPs, (v) which are preceded by, and occurring in parallel with, molecular changes involving determining sub‐cellular structures and functions; (vi) accompanied by progressive neuron loss and brain atrophy, (vii) prevalent in human brain aging, and (viii) manifested as pre‐clinical AD, and progressing not universally to mild cognitive impairment due to AD (MCI‐AD), and mild, moderate, and severe AD dementia (ADD).

**FIGURE 1:**
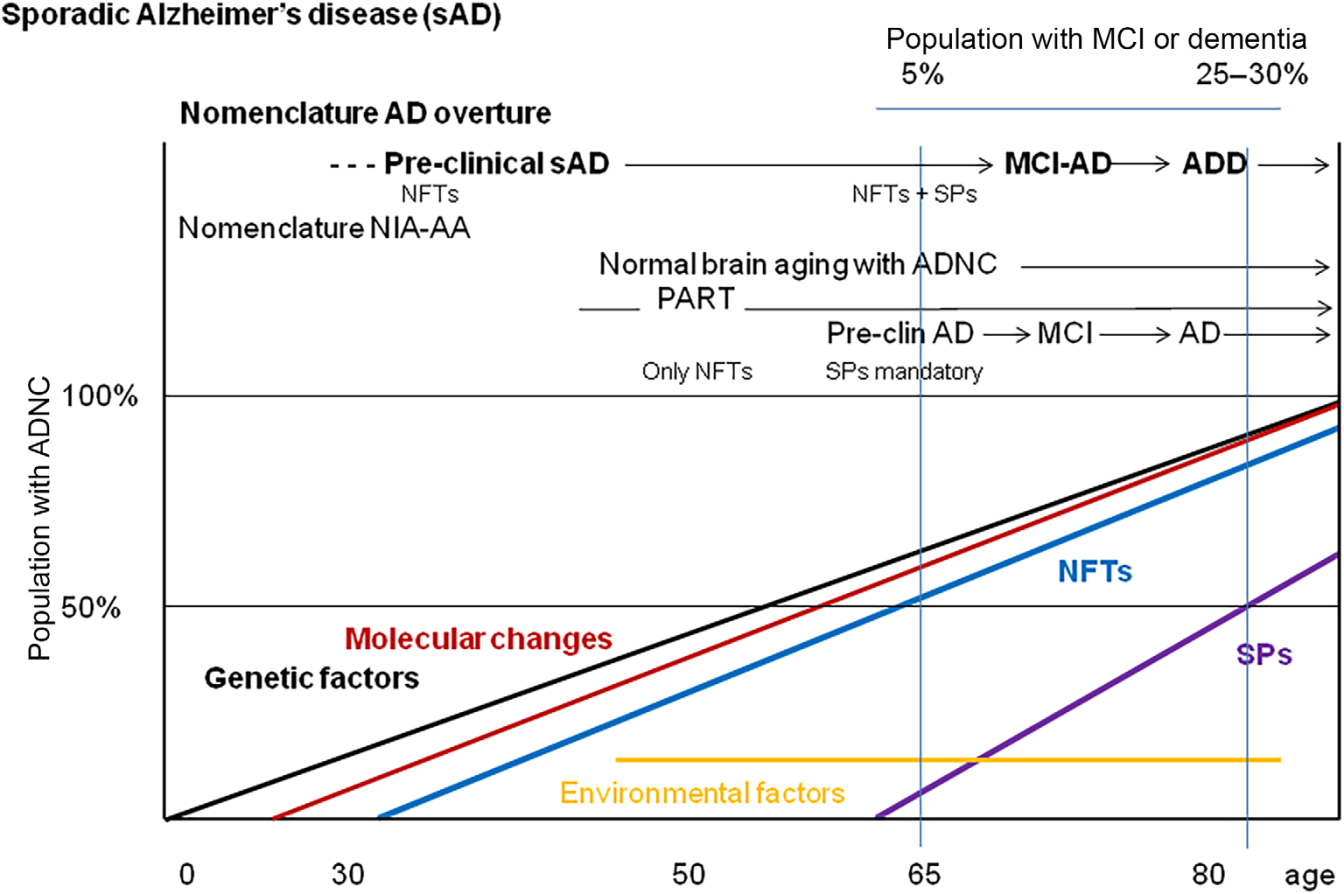
Schematic representation of the natural history of ADNC and associated genetic factors, environmental factors, molecular changes listed in Table 1, NFTs, and SPs with age in years. The proposed AD overture diagnosis and staging are compared with NIA‐AA guidelines. ADNC: Alzheimer's disease neuropathological changes; AD, Alzheimer's disease; ADD, AD dementia; MCI‐AD, mild cognitive impairment due to AD; NFTs, neurofibrillary tangles; PART, primary age‐related tauopathy; SPs, senile plaques. Major differences between NIA‐AA guidelines and AD overture are the consideration of tau pathology as the first ADNC marker of AD; pre‐clinical sAD is used instead of pre‐clinical AD; the diagnosis of pre‐clinical sAD is advanced by several decades based on the detection of tau pathology in the inner temporal cortex; normal brain aging with ADNC and PART are within the spectrum of sAD; molecular changes (most of them deserving in deep study) preceding, or occurring in parallel with, ADNC, converge at different times leading to neuronal and glial dysfunction, and act as inducers of ADNC; genetic factors have determining roles pointing to the relevance of lipid transport, membrane integrity, and neuroinflammation in the pathogenesis of sAD.

The neuropathological characteristics and clinical correlates of sAD overture compared with NIA‐AA guidelines are summarized in Figure 1.

The “ABC” score for ADNC proposed by the NIA‐AA is helpful, but Braak β‐amyloid scores are similar to CERAD neuritic plaque scores and might also be used to categorize the extension of plaques in the cerebral cortex. As a supplementary note, immunohistochemistry with validated antibodies against P‐tau and β‐amyloid is mandatory. 

The critical point that distinguishes the AD overture definition from the NIA‐AA proposal is the early appearance of tau pathology as the first neuropathological ADNC marker of sAD. The β‐amyloid cascade hypothesis is no longer considered the cause of sAD. However, the early appearance of tau pathology does not presuppose that tau pathology is the sAD's origin. As indicated in Figure 1, genetic factors and molecular changes summarized in Table 1 are considered the earliest and complementary partners that induce tau and β‐amyloid pathology at separate times of the lifespan.

Regarding the value of biomarkers suggested by the NIA‐AA, AD overture shifts to recognizing early tau pathology, mainly revealed by tau‐PET, as the earliest marker of sAD. However, new indicators are needed to detect early molecular alterations preceding, or occurring in parallel with, tau and β‐amyloid pathology. Among them, changes in protein and lipid composition of cell membranes, and altered membrane signaling with the cytoskeleton, mitochondria, and vesicles are significant targets for further research. Age‐related astroglial and microglial alterations, modification of neuroinflammatory profiles, and cerebral blood vessel dysfunction with age are determining, as well.

Finally, the clinical classification of AD overture also differs from the NIA‐AA classification in categorizing pre‐clinical sAD. Nevertheless, the terms MCI due to AD and mild, moderate, and severe Alzheimer's dementia are modified slightly in the new classification. MCI due to AD is named MCI‐AD, and mild, moderate, and severe Alzheimer's dementia, are named mild, moderate, and severe ADD. ADD is introduced to avoid confusion between AD (Alzheimer's disease) and AD dementia (restricted to the most advanced AD phase).

Regarding pre‐clinical sAD, AD overture guidelines propose that stage 1 corresponds to asymptomatic primary tauopathy as revealed with high‐resolution tau‐PET. Stages 2–4 are similar to stages 1–3, respectively, proposed by the NIA‐AA [83], but tau‐PET is positive in every stage. The new staging and diagnostic criteria for pre‐clinical sAD compared with the NIA‐AA guidelines are summarized in Table 2A, B.

**TABLE 2:** Comparison of staging categories for pre‐clinical AD between the NIA‐AA (A) and AD overture (B) guidelines

(A)				
Stage	Description	Aβ‐PET, low CSF Aβ_1–42_	High CSF tau/P‐tau, neuronal dysfunction (FDG‐PET or fMRI), cortical thinning, hippocampal atrophy (sMRI)	Subtle cognitive decline
1	Asymptomatic cerebral amyloidosis	Positive	Negative	Negative
2	Asymptomatic cerebral amyloidosis +“downstream” neurodegeneration	Positive	Positive	Negative
3	Cerebral amyloidosis +Neurodegeneration +Subtle cognitive decline	Positive	Positive	Positive

(B)					
Stage	Description	Tau‐PET	Aβ‐PET, low CSF Aβ_1–42_	High CSF tau/P‐tau, neuronal dysfunction (FDG‐PET or fMRI), hippocampal atrophy (sMRI)	Subtle cognitive decline
1	Asymptomatic primary tauopathy	Positive	Negative	Negative	Negative
2	+Asymptomatic cerebral amyloidosis	Positive	Positive	Negative	Negative
3	+Positive CSF tau biomarkers, +cerebral dysfunction	Positive	Positive	Positive	Negative
4	+subtle cognitive decline	Positive	Positive	Positive	Positive

Abbreviations: Aβ, β‐amyloid; FDG, fluorodeoxyglucose (18F); fMRI, functional magnetic resonance imaging; PET, positron emission tomography; sMRI, structural MRI.

The concept of human brain aging and sAD proposed in AD overture seems more closely adapted to the natural history of events during brain aging and sAD. AD overture has additional clinical implications. The diagnosis of pre‐clinical sAD has been advanced for several decades. Moreover, learning about molecular changes involving various structures and signaling pathways preceding or occurring in parallel with the appearance of tau pathology and β‐amyloid deposition may be an opportunity to design new unconventional, breakthrough therapies.

AD overture guidelines are centered on human brain aging and sAD. At present, we do not have enough information to apply the same parameters, particularly those linked to molecular brain changes at pre‐clinical stages and pre‐clinical biomarkers, to fAD caused by mutations in *APP*, *PSEN1*, and *PSEN2*.

## AUTHOR CONTRIBUTION

IF has written the paper.

## FUNDING INFORMATION

The project leading to these results received funding from the “la Caixa” Foundation under the agreement LCF/PR/HR19/52160007: HR18‐00452. I thank CIBERNED, Instituto de Salud Carlos III, and the CERCA programme of the Generalitat de Catalunya for institutional support.

## CONFLICT OF INTEREST

The authors declare no potential conflict of interest.

## Data Availability

No new data. All data are in the manuscript.

## References

[1] Alzheimer A . Über eine eigenartige Erkrankung der Hirnrinde. Allg Z Psychiat. 1907;64:146–8.

[2] Fischer O . Miliare Nekrosen mit drusigen Wucherungen der Neurofibrillen, eine regelmässige Veränderung der Hirnrinde bei seniler Demenz. Monatsschr Psychiat Neurol. 1907;22:361–72.

[3] Fischer O . Die presbyophrene Demenz, deren anatomische Grundlage und klinische Abgrenzung. Z ges Neurol Psychiat. 1910;3:371–471.

[4] McKhann G , Drachman D , Folstein M , Katzman R , Price D , Stadlan EM . Clinical diagnosis of Alzheimer's disease: report of the NINCDS‐ADRDA Work Group under the auspices of Department of Health and Human Services Task Force on Alzheimer's Disease. Neurology. 1984;34:939–44. 10.1212/wnl.34.7.939 6610841

[5] Price JL , Davis PB , Morris JC , White DL . The distribution of tangles, plaques and related immunohistochemical markers in healthy aging and Alzheimer's disease. Neurobiol Aging. 1991;12:295–312. 10.1016/0197-4580(91)90006-6 1961359

[6] Arriagada PV , Marzloff K , Hyman BT . Distribution of Alzheimer‐type pathological changes in nondemented elderly individuals matches the pattern in Alzheimer's disease. Neurology. 1992;42:1681–8. 10.1212/wnl.42.9.1681 1307688

[7] Bouras C , Hof PR , Morrison JH . Neurofibrillary tangle densities in the hippocampal formation in a non‐demented population define subgroups of patients with differential early pathologic changes. Neurosci Lett. 1993;153:131–5. 10.1016/0304-3940(93)90305-5 8327187

[8] Bouras C , Hof PR , Giannakopoulos P , Michel JP , Morrison JH . Regional distribution of neurofibrillary tangles and senile plaques in the cerebral cortex of elderly patients: a quantitative evaluation of a one‐year autopsy population from a geriatric hospital. Cereb Cortex. 1994;4:138–50. 10.1093/cercor/4.2.138 8038565

[9] Mirra SS , Heyman A , McKeel D , Sumi SM , Crain BJ , Brownlee LM , et al. The Consortium to Establish a Registry for Alzheimer's disease (CERAD). Part II. Standardization of the neuropathologic assessment of Alzheimer's disease. Neurology. 1991;41:479–86. 10.1212/wnl.41.4.479 2011243

[10] Mirra SS , Hart MN , Terry RD . Making the diagnosis of Alzheimer's disease. A primer for practicing pathologists. Arch Pathol Lab Med. 1993;117:132–44. 8427562

[11] Glenner GG , Wong CW . Alzheimer's disease: initial report of the purification and characterization of a novel cerebrovascular amyloid protein. Biochem Biophys Res Commun. 1984;120:885–90. 10.1016/j.bbrc.2012.08.020 6375662

[12] Glenner GG , Wong CW , Quaranta V , Eanes ED . The amyloid deposits in Alzheimer's disease: their nature and pathogenesis. Appl Pathol. 1984;12:357–69. 6242724

[13] Masters CL , Simms G , Weinman NA , Multhaup G , McDonald BL , Beyreuther K . Amyloid plaque core protein in Alzheimer disease and Down syndrome. Proc Natl Acad Sci USA. 1985;82:4245–9. 10.1073/pnas.82.12.4245 3159021 PMC397973

[14] Iwatsubo T , Odaka A , Suzuki N , Mizusawa H , Nukina N , Ihara Y . Visualization of a beta 42(43) and a beta 40 in senile plaques with end‐specific a beta monoclonals: evidence that an initially deposited species is a beta 42(43). Neuron. 1994;13:45–53. 10.1016/0896-6273(94)90458-8 8043280

[15] Delacourte A , Defossez A . Alzheimer's disease: tau proteins, the promoting factors of microtubule assembly, are major components of paired helical filaments. J Neurol Sci. 1986;76:173–86. 10.1016/0022-510x(86)90167-x 3098926

[16] Kosik KS , Joachim CL , Selkoe DJ . Microtubule‐associated protein tau (tau) is a major antigenic component of paired helical filaments in Alzheimer disease. Proc Natl Acad Sci USA. 1986;83:4044–8. 10.1073/pnas.83.11.4044 2424016 PMC323662

[17] Wood JG , Mirra SS , Pollock NJ , Binder LI . Neurofibrillary tangles of Alzheimer disease share antigenic determinants with the axonal microtubule‐associated protein tau (tau). Proc Natl Acad Sci USA. 1986;83:4040–3. 10.1073/pnas.83.11.4040 2424015 PMC323661

[18] Grundke‐Iqbal I , Iqbal K , Quinlan M , Tung YC , Zaidi MS , Wisniewski HM . Microtubule‐associated protein tau. A component of Alzheimer paired helical filaments. J Biol Chem. 1986;261:6084–9. 3084478

[19] Goedert M , Wischik CM , Crowther RA , Walker JE , Klug A . Cloning and sequencing of the cDNA encoding a core protein of the paired helical filament of Alzheimer disease: identification as microtubule‐associated protein tau. Proc Natl Acad Sci USA. 1988;85:4051–5. 10.1073/pnas.85.11.4051 3131773 PMC280359

[20] Goedert M , Spillantini MG , Cairns NJ , Crowther RA . Tau proteins in Alzheimer paired helical filaments: abnormal phosphorylation of all six brain isoforms. Neuron. 1992;8:159–68. 10.1016/0896-6273(92)90117-v 1530909

[21] Buée L , Bussière T , Buée‐Scherrer V , Delacourte A , Hof PR . Tau protein isoforms, phosphorylation and role in neurodegenerative disorders. Brain Res Brain Res Rev. 2000;33:95–130. 10.1016/s0165-0173(00)00019-9 10967355

[22] Mandelkow EM , Mandelkow E . Biochemistry and cell biology of tau protein in neurofbrillary degeneration. Cold Spring Harb Perspect Med. 2012;2:a006247. 22762014 10.1101/cshperspect.a006247 PMC3385935

[23] Spillantini MG , Goedert M . Tau pathology and neurodegeneration. Lancet Neurol. 2013;12:609–22. 10.1016/s0166-2236(98)01337-x 23684085

[24] Arendt T , Stieler JT , Holzer M . Tau and tauopathies. Brain Res Bull. 2016;26:238–92. 10.1016/j.brainresbull.2016.08.018 27615390

[25] Goedert M , Spillantini MG . Ordered assembly of tau protein and neurodegeneration. Adv Exp Med Biol. 2019;1184:3–21. 10.1007/978-981-32-9358-8_1 32096024

[26] Hernández F , Ferrer I , Pérez M , Zabala JC , Del Rio JA , Avila J . Tau aggregation. Neuroscience. 2022. 10.1016/j.neuroscience.2022.04.024 35525497

[27] Goate A , Chartier‐Harlin MC , Mullan M , Brown J , Crawford F , Fidani L , et al. Segregation of a missense mutation in the amyloid precursor protein gene with familial Alzheimer's disease. Nature. 1991;349:704–6. 10.1038/349704a0 1671712

[28] Chartier‐Harlin MC , Crawford F , Houlden H , Warren A , Hughes D , Fidani L , et al. Early‐onset Alzheimer's disease caused by mutations at codon 717 of the β‐amyloid precursor protein gene. Nature. 1991;353:844–6. 10.1038/353844a0 1944558

[29] Murrell J , Farlow M , Ghetti B , Benson MD . A mutation in the amyloid precursor protein associated with hereditary Alzheimer's disease. Science. 1991;254:97–9. 10.1126/science.1925564 1925564

[30] Levy‐Lahad E , Wasco W , Poorkaj P , Romano DM , Oshima J , Pettingell WH , et al. Candidate gene for the chromosome 1 familial Alzheimer's disease locus. Science. 1995;18(269):973–7. 10.1126/science.7638622 7638622

[31] Sherrington R , Rogaev EI , Liang Y , Rogaeva EA , Levesque G , Ikeda M , et al. Cloning of a gene bearing missense mutations in early‐onset familial Alzheimer's disease. Nature. 1995;375:754–60. 10.1038/375754a0 7596406

[32] Rogaev EI , Sherrington R , Rogaeva EA , Levesque G , Ikeda M , Liang Y , et al. Familial Alzheimer's disease in kindreds with missense mutations in a gene on chromosome 1 related to the Alzheimer's disease type 3 gene. Nature. 1995;376:775–8. 10.1038/376775a0 7651536

[33] Hardy JA , Higgins GA . Alzheimer's disease: the amyloid cascade hypothesis. Science. 1992;256:184–5. 10.1126/science.1566067 1566067

[34] Selkoe DJ , Hardy J . The amyloid hypothesis of Alzheimer's disease at 25 years. EMBO Mol Med. 2016;8:595–608. 10.15252/emmm.201606210 27025652 PMC4888851

[35] Cline EN , Bicca MA , Viola KL , Klein WL . The amyloid‐β oligomer hypothesis: beginning of the third decade. J Alzheimers Dis. 2018;64:S567–610. 10.3233/JAD-179941 29843241 PMC6004937

[36] Kalaria R , Ferrer I , Love S . Vascular disease, hypoxia and related conditions. In: Love S , Budka H , Ironside JW , Perry A , editors. Greenfield's Neuropathology. Volume 1. Boca Raton, London, New York: CRC Press, Taylor and Francis Group; 2015. p. 59–209.

[37] Zhang X , Li Y , Xu H , Zhang YW . The *γ*‐secretase complex: from structure to function. Front Cell Neurosci. 2014;8:427. 10.3389/fncel.2014.00427 25565961 PMC4263104

[38] Wolfe MS . Structure and function of the γ‐secretase complex. Biochemistry. 2019;58:2953–66. 10.1021/acs.biochem.9b00401 31198028 PMC6618299

[39] Halliday GM , Song YJ , Lepar G , Brooks WS , Kwok JB , Kersaitis C , et al. Pick bodies in a family with presenilin‐1 Alzheimer's disease. Ann Neurol. 2005;57:139–43. 10.1002/ana.20366 15622541

[40] Riudavets MA , Bartoloni L , Troncoso JC , Pletnikova O , St George‐Hyslop PS , Schultz M , et al. Familial dementia with frontotemporal features associated with M146V presenilin‐1 mutation. Brain Pathol. 2013;23:595–600. 10.1111/bpa.12051 23489366 PMC4007155

[41] Dermaut B , Kumar‐Singh S , Engelborghs S , Theuns J , Rademakers R , Saerens J , et al. A novel presenilin 1 mutation associated with pick bodies but not beta‐amyloid plaques. Ann Neurol. 2004;55:617–26. 10.1002/ana.20083 15122701

[42] Muratore CR , Rice HC , Srikanth P , Callahan DG , Shin T , Benjamin LN , et al. The familial Alzheimer's disease APPV717I mutation alters APP processing and Tau expression in iPSC‐derived neurons. Hum Mol Genet. 2014;23:3523–36. 10.1093/hmg/ddu064 24524897 PMC4049307

[43] Yagi T , Ito D , Okada Y , Akamatsu W , Nihei Y , Yoshizaki T , et al. Modeling familial Alzheimer's disease with induced pluripotent stem cells. Hum Mol Genet. 2011;20:4530–9. 10.1093/hmg/ddr394 21900357

[44] Gonzalez C , Armijo E , Bravo‐Alegria J , Becerra‐Calixto A , Mays CE , Soto C . Modeling amyloid beta and tau pathology in human cerebral organoids. Mol Psychiatry. 2018;23:2363–74. 10.1038/s41380-018-0229-8 30171212 PMC6594704

[45] Zhao J , Fu Y , Yamazaki Y , Ren Y , Davis MD , Liu CC , et al. APOE4 exacerbates synapse loss and neurodegeneration in Alzheimer's disease patient iPSC‐derived cerebral organoids. Nat Commun. 2020;11:5540. 10.1038/s41467-020-19264-0 33139712 PMC7608683

[46] Corder EH , Saunders AM , Strittmatter WJ , Schmechel DE , Gaskell PC , Small GW , et al. Gene dose of apolipoprotein E type 4 allele and the risk of Alzheimer's disease in late onset families. Science. 1993;261:921–3. 10.1126/science.8346443 8346443

[47] Saunders AM , Strittmatter WJ , Schmechel D , St George‐Hyslop PH , Pericak‐Vance MA , Joo SH . Association of apolipoprotein E allele epsilon 4 with late‐onset familial and sporadic Alzheimer's disease. Neurology. 1993;43:1467–72. 10.1212/wnl.43.8.1467 8350998

[48] Strittmatter WJ , Saunders AM , Schmechel D , Pericak‐Vance M , Enghild J , Salvesen GS . Apolipoprotein E: high‐avidity binding to beta‐amyloid and increased frequency of type 4 allele in late‐onset familial Alzheimer disease. Proc Natl Acad Sci USA. 1993;90:1977–81. 10.1073/pnas.90.5.1977 8446617 PMC46003

[49] Jun G , Naj AC , Beecham GW , Wang LS , Buros J , Gallins PJ , et al. Meta‐analysis confirms CR1, CLU, and PICALM as Alzheimer disease risk loci and reveals interactions with APOE genotypes. Arch Neurol. 2010;67:1473–84. 10.1001/archneurol.2010.201 20697030 PMC3048805

[50] Jones L , Holmans PA , Hamshere ML , Harold D , Moskvina V , Ivanov D , et al. Genetic evidence implicates the immune system and cholesterol metabolism in the aetiology of Alzheimer's disease. PLoS One. 2010;5:e13950. 10.1371/journal.pone.0013950 21085570 PMC2981526

[51] Lambert JC , Ibrahim‐Verbaas CA , Harold D , Naj AC , Sims R , Bellenguez C , et al. Meta‐analysis of 74,046 individuals identifies 11 new susceptibility loci for Alzheimer's disease. Nat Genet. 2013;45:1452–8. 10.1038/ng.2802 24162737 PMC3896259

[52] Sims R , van der Lee SJ , Naj AC , Bellenguez C , Badarinarayan N , Jakobsdottir J , et al. Rare coding variants in PLCG2, ABI3, and TREM2 implicate microglial‐mediated innate immunity in Alzheimer's disease. Nat Genet. 2017;49:1373–84. 10.1038/ng.3916 28714976 PMC5669039

[53] Tansey KE , Cameron D , Hill MJ . Genetic risk for Alzheimer's disease is concentrated in specific macrophage and microglial transcriptional networks. Genome Med. 2018;10:14. 10.1186/s13073-018-0523-8 29482603 PMC5828245

[54] Jansen IE , Savage JE , Watanabe K , Bryois J , Williams DM , Steinberg S , et al. Genome‐wide meta‐analysis identifies new loci and functional pathways influencing Alzheimer's disease risk. Nat Genet. 2019;51:404–13. 10.1038/s41588-018-0311-9 30617256 PMC6836675

[55] Bellenguez C , Küçükali F , Jansen IE , Kleineidam L , Moreno‐Grau S , Amin N , et al. New insights into the genetic etiology of Alzheimer's disease and related dementias. Nat Genet. 2022;54:412–36. 10.1038/s41588-022-01024-z 35379992 PMC9005347

[56] Morgan K . The three new pathways leading to Alzheimer's disease. Neuropathol Appl Neurobiol. 2011;37:353–7. 10.1111/j.1365-2990.2011.01181.x 21486313

[57] Braak H , Braak E . Neuropathological stageing of Alzheimer‐related changes. Acta Neuropathol. 1991;82:239–59. 10.1007/BF00308809 1759558

[58] Braak H , Braak E . Staging of Alzheimer's disease‐related neurofibrillary changes. Neurobiol Aging. 1995;16:271–8. 10.1016/0197-4580(95)00021-6 7566337

[59] Braak H , Braak E . Frequency of stages of Alzheimer‐related lesions in different age categories. Neurobiol Aging. 1997;18:351–7. 10.1016/s0197-4580(97)00056 9330961

[60] Braak H , Del Tredici K . The pathological process underlying Alzheimer's disease in individuals under thirty. Acta Neuropathol. 2011;121:171–81. 10.1007/s00401-010-0789-4 21170538

[61] Arnsten AFT , Datta D , Del Tredici K , Braak H . Hypothesis: Tau pathology is an initiating factor in sporadic Alzheimer's disease. Alzheimer‘s Dement. 2021;17:115–24. 10.1002/alz.12192 33075193 PMC7983919

[62] Grinberg LT , Rüb U , Ferretti RE , Nitrini R , Farfel JM , Polichiso L , et al. The dorsal raphe nucleus shows phospho‐tau neurofibrillary changes before the transentorhinal region in Alzheimer's disease. A precocious onset? Neuropathol Appl Neurobiol. 2009;35:406–16. 10.1111/j.1365-2990.2009.00997.x 19508444

[63] Braak H , Del Tredici K . The preclinical phase of the pathological process underlying Alzheimer's disease. Brain. 2015;138:2814–33. 10.1093/brain/awv236 26283673

[64] Arendt T , Bruckner MK , Morawski M , Jager C , Gertz HJ . Early neurone loss in Alzheimer's disease: cortical or subcortical? Acta Neuropathol Commun. 2015;3(10). 10.1186/s40478-015-0187-1 PMC4359478 25853173

[65] Andrés‐Benito P , Fernández‐Dueñas V , Carmona M , Escobar LA , Torrejón‐Escribano B , Aso E , et al. Locus coeruleus at asymptomatic early and middle Braak stages of neurofibrillary tangle pathology. Neuropathol Appl Neurobiol. 2017;43:373–92. 10.1111/nan.12386 28117912

[66] Šimić G , Babić Leko M , Wray S , Harrington CR , Delalle I , Jovanov‐Milošević N , et al. Monoaminergic neuropathology in Alzheimer's disease. Prog Neurobiol. 2017;151:101–38. 10.1016/j.pneurobio.2016.04.001 27084356 PMC5061605

[67] Thal DR , Rüb U , Orantes M , Braak H . Phases of Aβ‐deposition in the human brain and its relevance for the development of AD. Neurology. 2002;58:1791–800. 10.1212/WNL.58.12.1791 12084879

[68] Braak H , Thal DR , Ghebremedhin E , Del Tredici K . Stages of the pathologic process in Alzheimer disease: age categories from 1 to 100 years. J Neuropathol Exp Neurol. 2011;70:960–9. 10.1097/NEN.0b013e318232a379 22002422

[69] Ferrer I . Defining Alzheimer as a common age‐related neurodegenerative process not inevitably leading to dementia. Prog Neurobiol. 2012;97:38–51. 10.1016/j.pneurobio.2012.03.005 22459297

[70] Armstrong RA , Myers D , Smith CU . The spatial patterns of plaques and tangles in Alzheimer's disease do not support the ‘Cascade Hypothesis’. Dementia. 1993;4:16–20. 10.1159/000107291 8358502

[71] Herrup K . The case for rejecting the amyloid cascade hypothesis. Nat Neurosci. 2015;18:794–9. 10.1038/nn.4017 26007212

[72] Delacourte A , Sergeant N , Champain D , Wattez A , Maurage CA , Lebert F , et al. Nonoverlapping but synergetic tau and APP pathologies in sporadic Alzheimer's disease. Neurology. 2002;59:398–407. 10.1212/wnl.59.3.398 12177374

[73] Delacourte A . Alzheimer's disease: a true tauopathy fueled by amyloid precursor protein dysfunction. In: Hanin I , Cacabelos R , Fisher A , editors. Recent progress in Alzheimer's and Parkinson's diseases. New York: Taylor & Francis; 2005. p. 301–7.

[74] Crystal H , Dickson D , Fuld P , Crystal H , Dickson D , Fuld P , et al. Clinico‐pathologic studies in dementia: nondemented subjects with pathologically confirmed Alzheimer's disease. Neurology. 1988;38:1682–7. 10.1212/wnl.38.11.1682 3185902

[75] Knopman DS , Parisi JE , Salviati A , Floriach‐Robert M , Boeve BF , Ivnik RJ , et al. Neuropathology of cognitively normal elderly. J Neuropathol Exp Neurol. 2003;62:1087–95. 10.1093/jnen/62.11.1087 14656067

[76] Bennett DA , Schneider JA , Arvanitakis Z , Kelly JF , Aggarwal NT , Shah RC , et al. Neuropathology of older persons without cognitive impairment from two community‐based studies. Neurology. 2006;66:1837–44. 10.1212/01.wnl.0000219668.47116.e6 16801647

[77] Tsartsalis S , Aikaterini Xekardaki A , Hof PR , Kövari E , Bouras C . Early Alzheimer‐type lesions in cognitively normal subjects. Neurobiol Aging. 2018;62:34–44. 10.1016/j.neurobiolaging.2017.10.002 29107845 PMC5743763

[78] Hubbard BM , Fenton GW , Anderson JM . A quantitative histological study of early clinical and preclinical Alzheimer's disease. Neuropathol Appl Neurobiol. 1990;16:111–21. 10.1111/j.1365-2990.1990.tb00940.x 2345598

[79] Hulette C , Welsh‐Bohmer K , Murray M , Saunders A , Mash D , Mcintyre L . Neuropathological and neuropsychological changes in "normal" aging: evidence for preclinical Alzheimer disease in cognitively normal individuals. J Neuropathol Exp Neurol. 1998;57:1168–274. 10.1097/00005072-199812000-00009 9862640

[80] Price J , Morris JC . Tangles and plaques in non demented aging and "preclinical" Alzheimer's disease. Ann Neurol. 1999;45:358–68. 10.1002/1531-8249(199903)45:3<358::aid-ana12>3.0.co;2-x 10072051

[81] Hyman BT , Phelps CH , Beach TG , Bigio EH , Cairns NJ , Carrillo MC , et al. National Institute on Aging‐Alzheimer's Association disease guidelines for the neuropathologic assessment of Alzheimer's disease. Alzheimers Dement. 2012;8:1–13. 10.1016/j.jalz.2011.10.007 22265587 PMC3266529

[82] Montine TJ , Phelps CH , Beach TG , Bigio EH , Cairns NJ , Dickson DW , et al. National Institute on Aging‐Alzheimer's Association guidelines for the neuropathologic assessment of Alzheimer's disease: a practical approach. Acta Neuropathol. 2012;123:1–11. 10.1007/s00401-011-0910-3 22101365 PMC3268003

[83] Sperling RA , Aisen PS , Beckett LA , Bennett DA , Craft S , Fagan AM , et al. Toward defining the preclinical stages of Alzheimer's disease: recommendations from the National Institute on Aging‐Alzheimer's Association workgroups on diagnostic guidelines for Alzheimer's disease. Alzheimers Dement. 2011;7:280–92. 10.1016/j.jalz.2011.03.003 21514248 PMC3220946

[84] Olsson B , Lautner R , Andreasson U , Öhrfelt A , Portelius E , Bjerke M , et al. CSF and blood biomarkers for the diagnosis of Alzheimer's disease: a systematic review and meta‐analysis. Lancet Neurol. 2016;15:673–84. 10.1016/S1474-4422(16)00070-3 27068280

[85] Dubois B , Hampel H , Feldman HH , Scheltens P , Aisen P , Andrieu S , et al. Preclinical Alzheimer's disease: definition, natural history, and diagnostic criteria. Alzheimers Dement. 2016;12:292–323. 10.1016/j.jalz.2016.02.002 27012484 PMC6417794

[86] Blennow K , Zetterberg H . Biomarkers for Alzheimer's disease: current status and prospects for the future. J Intern Med. 2018;284:643–63. 10.1111/joim.12816 30051512

[87] Bjerke M , Engelborghs S . Cerebrospinal fluid biomarkers for early and differential Alzheimer's disease diagnosis. J Alzheimers Dis. 2018;62:1199–209. 10.3233/JAD-170680 29562530 PMC5870045

[88] Zetterberg H , Bendlin BB . Biomarkers for Alzheimer's disease‐preparing for a new era of disease‐modifying therapies. Mol Psychiatry. 2021;26:296–308. 10.1038/s41380-020-0721-9 32251378 PMC8172244

[89] Leuzy A , Cullen NC , Mattsson‐Carlgren N , Hansson O . Current advances in plasma and cerebrospinal fluid biomarkers in Alzheimer's disease. Curr Opin Neurol. 2021;34:266–74. 10.1097/WCO.0000000000000904 33470669

[90] Teunissen CE , Verberk IMW , Thijssen EH , Vermunt L , Hansson O , Zetterberg H , et al. Blood‐bassed biomarkers for Alzheimer's disease: towards clinical implementation. Lancet Neurol. 2022;21:66–77. 10.1016/S1474-4422(21)00361-6 34838239

[91] Jansen WJ , Ossenkoppele R , Knol DL , Tijms BM , Scheltens P , Verhey FR , et al. Prevalence of cerebral amyloid pathology in persons without dementia: a meta‐analysis. JAMA. 2015;313:1924–38. 10.1001/jama.2015.4668 25988462 PMC4486209

[92] Schöll M , Lockhart SN , Schonhaut DR , O'Neil JP , Janabi M , Ossenkoppele R , et al. PET imagingof tau deposition in the aging human brain. Neuron. 2016;89:971–82. 10.1016/j.neuron.2016.01.028 26938442 PMC4779187

[93] Mueller A , Bullich S , Barret O , Madonia J , Berndt M , Papin C , et al. Tau PET imaging with ^18^F‐PI‐2620 in patients with Alzheimer disease and healthy controls: a first‐in‐humans study. J Nucl Med. 2020;61:911–9. 10.2967/jnumed.119.236224 31712323 PMC7262222

[94] Jack CR , Wiste HJ , Weigand SD , Therneau TM , Knopman DS , Lowe V , et al. Age‐specific and sex‐specific prevalence of cerebral β‐amyloidosis, tauopathy, and neurodegeneration in cognitively unimpaired individuals aged 50–95 years: a cross‐sectional study. Lancet Neurol. 2017;16:435–44. 10.1016/S1474-4422(17)30077-7 28456479 PMC5516534

[95] Lowe VJ , Wiste HJ , Senjem ML , Weigand SD , Therneau TM , Boeve BF , et al. Widespread brain tau and its association with ageing, Braak stage and Alzheimer's dementia. Brain. 2018;141:271–87. 10.1093/brain/awx320 29228201 PMC5837250

[96] Yoon B , Guo T , Provost K , Korman D , Ward TJ , Landau SM , et al. Abnormal tau in amyloid PET negative individuals. Neurobiol Aging. 2022;109:125–34. 10.1016/j.neurobiolaging.2021.09.019 34715443 PMC9695003

[97] Berron D , Vogel JW , Insel PS , Pereira JB , Xie L , Wisse LEM , et al. Early stages of tau pathology and its associations with functional connectivity, atrophy and memory. Brain. 2021;144:2771–83. 10.1093/brain/awab114 33725124 PMC8557349

[98] Sanchez JS , Becker JA , Jacobs HIL , Hanseeuw BJ , Jiang S , Schultz AP , et al. The cortical origin and initial spread of medial temporal tauopathy in Alzheimer's disease assessed with positron emission tomography. Sci Transl Med. 2021;13:eabc0655. 10.1126/scitranslmed.abc0655 33472953 PMC7978042

[99] Wisse LEM , Xie L , Das SR , De Flores R , Hansson O , Habes M , et al. Tau pathology mediates age effects on medial temporal lobe structure. Neurobiol Aging. 2022;109:135–44. 10.1016/j.neurobiolaging.2021.09.017 34740075 PMC8800343

[100] Ossenkoppele R , Smith R , Mattsson‐Carlgren N , Groot C , Leuzy A , Strandberg O , et al. Accuracy of tau positron emission tomography as a prognostic marker in preclinical and prodromal Alzheimer disease: a head‐to‐head comparison against amyloid positron emission tomography and magnetic resonance imaging. JAMA Neurol. 2021;78:961–71. 10.1001/jamaneurol.2021.1858 34180956 PMC8240013

[101] Dubois B , Feldman HH , Jacova C , Cummings JL , Dekosky ST , Barberger‐Gateau P , et al. Revising the definition of Alzheimer's disease: a new lexicon. Lancet Neurol. 2010;9:1118–27. 10.1016/S1474-4422(10)70223-4 20934914

[102] Albert MS , Dekosky ST , Dickson D , Dubois B , Feldman HH , Fox NC , et al. The diagnosis of mild cognitive impairment due to Alzheimer's disease: recommendations from the National Institute on Aging‐Alzheimer's Association workgroups on diagnostic guidelines for Alzheimer's disease. Alzheimers Dement. 2011;7:270–9. 10.1016/j.jalz.2011.03.008 21514249 PMC3312027

[103] McKhann GM , Knopman DS , Chertkow H , Hyman BT , Jack CR Jr , Kawas CH , et al. The diagnosis of dementia due to Alzheimer's disease: recommendations from the National Institute on Aging‐Alzheimer's Association workgroups on diagnostic guidelines for Alzheimer's disease. Alzheimers Dement. 2011;7:263–9. 10.1016/j.jalz.2011.03.005 21514250 PMC3312024

[104] Ward A , Tardiff S , Dye C , Arrighi HM . Rate of conversion from prodromal Alzheimer's disease to Alzheimer's dementia: a systematic review of the literature. Dement Geriatr Cogn Disord Extra. 2013;3:320–32. 10.1159/000354370 PMC3808216 24174927

[105] Petersen RC , Lopez O , Armstrong MJ , Getchius TSD , Ganguli M , Gloss D , et al. Practice guideline update summary: mild cognitive impairment. Neurology. 2018;90:126–35. 10.1212/WNL.0000000000004826 29282327 PMC5772157

[106] Jack CR , Bennett DA , Blennow K , Carrillo MC , Dunn B , Haeberlein SB , et al. NIA‐AA research framework: toward a biological definition of Alzheimer's disease. Alzheimers Dement. 2018;14:535–62. 10.1016/j.jalz.2018.02.018 29653606 PMC5958625

[107] Nelson PT , Alafuzoff I , Bigio EH , Bouras C , Braak H , Cairns NJ , et al. Correlation of Alzheimer disease neuropathologic changes with cognitive status: a review of the literature. J Neuropathol Exp Neurol. 2012;71:362–81. 10.1097/NEN.0b013e31825018f7 22487856 PMC3560290

[108] Crary JF , Trojanowski JQ , Schneider JA , Abisambra JF , Abner EL , Alafuzoff I , et al. Primary age‐related tauopathy (PART): a common pathology associated with human aging. Acta Neuropathol. 2014;128:755–66. 10.1007/s00401-014-1349-0 25348064 PMC4257842

[109] Jellinger KA , Alafuzoff I , Attems J , Beach TG , Cairns NJ , Crary JF , et al. PART, a distinct tauopathy, different from classical sporadic Alzheimer disease. Acta Neuropathol. 2015;129:757–62. 10.1007/s00401-015-1407-2.E 25778618 PMC4534004

[110] Bell WR , An Y , Kageyama Y , English C , Rudow GL , Pletnikova O , et al. Neuropathologic, genetic, and longitudinal cognitive profiles in primary age‐related tauopathy (PART) and Alzheimer's disease. Alzheimers Dement. 2019;15:8–16. 10.1016/j.jalz.2018.07.215 30465754 PMC6542566

[111] Teylan M , Mock C , Gauthreaux K , Chen YC , Chan KCG , Hassenstab J , et al. Cognitive trajectory in mild cognitive impairment due to primary age‐related tauopathy. Brain. 2020;143:611–21. 10.1093/brain/awz403 31942622 PMC7009602

[112] Jellinger KA , Bancher C . Senile dementia with tangles (tangle predominant form of senile dementia). Brain Pathol. 1998;8:367–76. 10.1111/j.1750-3639.1998.tb00160.x 9546293 PMC8098213

[113] McMillan CT , Lee EB , Jefferson‐George K , Naj A , Van Deerlin VM , Trojanowski JQ , et al. Alzheimer's genetic risk is reduced in primary age‐related tauopathy: a potential model of resistance? Ann Clin Transl Neurol. 2018;5:927–34. 10.1002/acn3.581 30128317 PMC6093846

[114] Robinson AC , Davidson YS , Roncaroli F , Minshull J , Tinkler P , Horan MA , et al. Influence of APOE genotype in primary age‐related tauopathy. Acta Neuropathol Commun. 2020;8:215. 10.1186/s40478-020-01095-1 33287896 PMC7720601

[115] Santa‐Maria I , Varghese M , Ksiéżak‐Reding H , Dzhun A , Wang J , Pasinetti GM . Paired helical filaments from Alzheimer disease brain induces intracellular accumulation of tau protein in aggresomes. J Biol Chem. 2012;287:20522–33. 10.1074/jbc.M111.323279 22496370 PMC3370237

[116] Duyckaerts C , Braak H , Brion JP , Buée L , Del Tredici K , Goedert M , et al. PART is part of Alzheimer's disease. Acta Neuropathol. 2015;129:749–56. 10.1007/s00401-015-1390-7 25628035 PMC4405349

[117] Hickman RA , Flowers XE , Wisniewski T . Primary age‐related Tauopathy (PART): addressing the spectrum of neuronal tauopathic changes in the aging brain. Curr Neurol Neurosci Rep. 2020;20:39. 10.1007/s11910-020-01063-1 32666342 PMC7849162

[118] Arendt T . Alzheimer's disease as a disorder of dynamic brain self‐organization. Prog Brain Res. 2005;147:355–78. 10.1016/S0079-6123(04)47025-3 15581717

[119] De Strooper B , Karran E . The cellular phase of Alzheimer's disease. Cell. 2016;164:603–15. 10.1016/j.cell.2015.12.056 26871627

[120] Ferrer I . Alzheimer's disease is an inherent, natural part of human brain aging: an integral perspective. Free Neuropathol. 2022;3(17). 10.17879/freeneuropathology-2022-3806 PMC10209894 37284149

[121] Spires‐Jones TL , Attems J , Thal DR . Interactions of pathological proteins in neurodegenerative diseases. Acta Neuropathol. 2017;134:187–205. 10.1007/s00401-017-1709-7 28401333 PMC5508034

